# Factors affecting fungus-induced larval mortality in *Anopheles gambiae *and *Anopheles stephensi*

**DOI:** 10.1186/1475-2875-9-22

**Published:** 2010-01-19

**Authors:** Tullu Bukhari, Anthonieke Middelman, Constantianus JM Koenraadt, Willem Takken, Bart GJ Knols

**Affiliations:** 1 Laboratory of Entomology, Wageningen University, Wageningen, the Netherlands; 2 Division of Infectious Diseases, Tropical Medicine & AIDS, Academic Medical Center, University of Amsterdam, the Netherlands

## Abstract

**Background:**

Entomopathogenic fungi have shown great potential for the control of adult malaria vectors. However, their ability to control aquatic stages of anopheline vectors remains largely unexplored. Therefore, how larval characteristics (*Anopheles *species, age and larval density), fungus (species and concentration) and environmental effects (exposure duration and food availability) influence larval mortality caused by fungus, was studied.

**Methods:**

Laboratory bioassays were performed on the larval stages of *Anopheles gambiae *and *Anopheles stephensi *with spores of two fungus species, *Metarhizium anisopliae *and *Beauveria bassiana*. For various larval and fungal characteristics and environmental effects the time to death was determined and survival curves established. These curves were compared by Kaplan Meier and Cox regression analyses.

**Results:**

*Beauveria bassiana *and *Metarhizium anisopliae *caused high mortality of *An. gambiae *and *An. stephensi *larvae. However, *Beauveria bassiana *was less effective (Hazard ratio (HR) <1) compared to *Metarhizium anisopliae. Anopheles stephensi *and *An. gambiae *were equally susceptible to each fungus. Older larvae were less likely to die than young larvae (HR < 1). The effect of increase in fungus concentration on larval mortality was influenced by spore clumping. One day exposure to fungal spores was found to be equally effective as seven days exposure. In different exposure time treatments 0 - 4.9% of the total larvae, exposed to fungus, showed infection at either the pupal or adult stage. Mortality rate increased with increasing larval density and amount of available food.

**Conclusions:**

This study shows that both fungus species have potential to kill mosquitoes in the larval stage, and that mortality rate depends on fungus species itself, larval stage targeted, larval density and amount of nutrients available to the larvae. Increasing the concentration of fungal spores or reducing the exposure time to spores did not show a proportional increase and decrease in mortality rate, respectively, because the spores clumped together. As a result spores did not provide uniform coverage over space and time. It is, therefore, necessary to develop a formulation that allows the spores to spread over the water surface. Apart from formulation appropriate delivery methods are also necessary to avoid exposing non-target organisms to fungus.

## Background

Over the last decade, the potential of larval control has been increasingly recognized in the realm of integrated malaria control programmes [1-4]. Larval control can be economical and effective when applied with a good understanding of local disease determinants and vector ecology [5-7]. In urban areas, the breeding sites of mosquitoes are well defined and accessible, making it easier to target them. As a result larval control can steadily support other intervention methods like indoor residual spraying (IRS) and/or insecticide-treated bed nets (ITNs) [8]. Increasing urbanization thus underscores the need to develop larval control methods [7,9,10]. In this perspective, *Bacillus thuringiensis *var. *israelensis *(*Bti*) and *B. sphaericus *(*Bs*) have been applied on a large scale in urban settings and provided promising results [8,11]. In general, there is relatively little chance of resistance developing against these biological control agents as compared to chemical-based interventions like IRS and ITNs although it cannot be ruled out completely. For instance, the development of resistance against *Bs *in *Culex *spp. was reported within two years of field application [12,13]. It is, therefore, worthwhile to investigate other biological control agents for their larvicidal properties in order to extend the existing arsenal of larval biopesticides.

The entomopathogenic characteristic of *Metarhizium anisopliae *was identified more than 125 years ago while that of *Beauveria bassiana *was noted even earlier, in 1835 [14]. These entomopathogens belong to a group of anamorphic fungi called Hyphomycetes, which reproduce by spores (conidia) [15]. The spores have proven effective against mosquito larvae of the genus *Aedes, Culex *and *Anopheles *in the laboratory [16-20]. Studies have been undertaken to increase the virulence of the spores by insect-passaging and to increase the persistence of the spores through formulation [21,22]. Miranpuri and Khachatourians [23] showed that the primary infection sites of the fungal spores in the larval bodies is the head and anal region while fungal development mostly takes place in the larval gut. Apart from the larval stage, eggs (*Aedes*) treated with *M. anisopliae *and *B. bassiana *show a reduced hatch rate. Ovicidal property of *M. anisopliae *is best expressed at high humidity, which is a normal characteristic of anopheline oviposition sites [24,25]. However, attempts to infect newly emerged adults by dusting spores on vegetation surrounding the breeding sites were unsuccessful under semi-field conditions [17]. Scholte *et al *[26] infected adult mosquitoes by providing them with a fungus-treated resting site in the form of fungus-treated black cloth attached to ceilings of rural houses in Tanzania [26]. Farenhorst *et al *showed how water storage pots can serve as a suitable site for delivering a fungal infection to resting adult mosquitoes [27,28].

Use of fungal spores as a larvicide could complement adult control but in areas where the breeding sites are well defined, accessible and are not being used for domestic purposes. A number of factors can influence larval mortality caused by fungus, e.g. species and larval stage of mosquito targeted, besides the species, isolate and amount of the fungus applied. As in field the persistence of the spores can be affected by environmental conditions, mosquitoes may not be exposed to virulent spores for long. Reduced exposure time can also influence the control potential of fungus. Even without a control agent, the amount of nutrients in the breeding sites and larval density is known to have impact on larval survival [29]. Availability of nutrients can also decrease the intake of fungal spores by larvae, as fungal spores mainly enter the larval gut, the main infection site, due to ingestion [30]. These factors can increase or decrease the impact of fungal spores on the larvae. In this laboratory-based study the effect of the above-mentioned factors was evaluated on fungus-induced larval mortality for a better comprehension of its control potential and scope for field application. Two entomopathogenic fungi, *M. anisopliae *and *B. bassiana*, were tested against *An. stephensi *and *An. gambiae. Anopheles stephensi *is the main Asian malaria vector that breeds predominantly in man-made habitats while *An. gambiae *is an important African malaria vector that breeds in temporary aquatic habitats.

## Methods

### Mosquitoes

*Anopheles stephensi *(Strain STE 2, MRA no. 128, origin India) and *An. gambiae s.s*. (Suakoko strain, courtesy of Prof. M. Coluzzi) were reared in climate controlled chambers maintained at a temperature of 27 ± 1°C, 12L:12D photoperiod and a relative humidity of 70 ± 5%. The adults, kept in holding cages (30 × 30 × 30 cm), had *ad libitum *access to 6% glucose/water solution. 4-6 Days old females were blood fed on the forearm of a volunteer and provided with oviposition cups, covered with a cone-shaped filter paper. The eggs laid on the filter paper were transferred to plastic trays (25 × 25 × 8 cm). Hatched larvae were fed on Liquifry No. 1 (Interpet Ltd., Dorking, Surrey, UK) for the first two days and then on Tetramin^® ^for the rest of the larval period. Pupae were collected in small cups and transferred to holding cages.

### Fungus spores

Spores of *Metarhizium anisopliae *(ICIPE-30) and *Beauveria bassiana *(IMI- 391510) were provided by the Department of Bioprocess Engineering, Wageningen University and stored in plastic tubes at 4°C.

### Experimental conditions

All experiments were performed in rooms with climatic conditions similar to the rearing chambers. Tap water was left overnight in the plastic larval trays for dechlorination.

### Susceptibility of mosquito species, the effect of fungus species, larval stage and fungus concentration

It was determined whether larvae are more or less likely to be infected with fungal spores depending on their species, development stage, fungus species and spore concentration. This was done by comparing the mortality of early (L_1-2_, 1-3 days old) and late (L_3-4_, 4-8 days old) larval stages of *An. stephensi *and *An. gambiae *caused by different concentrations of *M. anisopliae *or *B. bassiana *spores. Fifty (L_1-2 _or L_3-4_) larvae were placed in plastic trays (25 × 25 × 8 cm), filled with 1 litre of dechlorinated water, and fed on Tetramin^® ^(0.1-0.2 mg/larva/day for L_1-2 _and 0.3 mg/larva/day for L_3-4_) [31]. In the treatment trays fungal spores were dusted on the water surface (441 cm^2^) in different amounts i.e. 2.5, 5, 10 or 20 mg. Dead larvae and pupae were recorded daily and removed for the next 12 days. The amount of food added was adjusted to the daily mortality and/or pupation. The resulting 40 treatments (two mosquito species × two developmental stages × two fungus species × four fungal concentrations and one control) were each replicated four times. The treatments and the replicates were not run in parallel but at different times depending upon the availability of experimental space and mosquito larvae. Every replicate had a control and the four fungus concentrations. The same protocol was followed each time and the same batch of fungi was used for all these experiments.

### Effect of exposure time

The mortality of L_3-4 _larvae of *An. stephensi *and *An. gambiae *when exposed to *M. anisopliae *or *B. bassiana *spores for different time periods was compared. Fifty larvae were placed in plastic trays and fed as described above. Out of five trays, four were treated with 10 mg of spores and one was used as control. After one day, the larvae that had not died or pupated, in a fungus-treated tray were transferred by a plastic pipette to a clean water tray, with an intermediate rinsing to reduce the chance of transferring spores. Similar transfers were carried out after 3, 5, and 7 days. The number of larvae that could be transferred on these days was less than 50 because larvae either pupated or died due to fungus infection. The control larvae were also transferred to compensate for any mechanical injury caused by the pipette. The control larvae were transferred to an another tray containing fresh water on day 1. This day was selected because at that time most of the larvae were still alive. Prior to and after the transfer, the larvae were fed (0.3 mg/larva/day) and monitored over 10 days, for death or pupation. All the trays, the untreated and treated water trays in which the larvae were added first, the trays that were used for rinsing the larvae and the trays, into which the larvae were transferred, were all filled with water at the same time. This was to ensure that when a larva was transferred from one tray to another the only difference was the absence of fungus and not fresh acclimatized water. The resulting pupae were kept in a plastic cup for adult emergence. The wing length (right wing, ventral side, from the notch of the alula to the wing tip) of the adults was measured (in mm), to the nearest second decimal place, as a proxy for mosquito body size [32]. The 20 treatments (two mosquito species × two fungal species × four time periods and one control) were each replicated four times. The pupae and adults that showed fungus infection, apparent due to mycelia growth, were observed under a microscope to identify the fungus species [15]. Fungus species was identified to make sure that the infection was due to *M. anisopliae *or *B. bassiana*. Similar to the first experiment the replicates and treatments were not carried out in parallel. Every replicate consisted of a control and the four exposure times. The same protocol was used for each replica and treatment. Further the same batch of fungi was used for all the treatments and replicates.

### Effect of food quantity

This experiment was carried out to determine if a lower quantity of food will increase the intake of fungal spores and lead to an increased mortality rate. In separate trays, L_3-4 _larvae were exposed to the same amount of fungal spores but provided with two different quantities of food or no food at all. The quantities were based on the study by Koenraadt *et al *[29] in which it was shown that 0.5 mg/larva/day adversely affected larval survival. A lower quantity was 0.3 mg/larva/day which is the standard quantity of food provided to L_3-4 _larvae in the laboratory. Each replica consisted of six trays. Fifty larvae were added to each tray. Three trays were treated with 10 mg of fungal spores, while the rest served as control. Larvae in one treated and one control tray were provided with food at a rate of 0.5 mg/larva/day (F_1_). Similarly larvae in another control and treated tray were provided with 0.3 mg/larva/day (F_2_) of food while those in the last two trays had no food (F_3_) at all. Food here refers to Tetramin^®^. Both mosquito and fungus species were tested. The mortality and/or pupation was recorded for 10 days. The experiment was replicated four times. For each mosquito, all the treatments and replicates were carried out in parallel.

### Effect of larval density

To determine the effect of larval density on fungal induced larval mortality, 50, 150 and 250 L_3-4 _larvae were added to plastic trays. This resulted in three larval densities i.e. 0.1 (D_1_), 0.3 (D_2_) and 0.5 (D_3_) larvae/cm^2 ^[29]. Apart from the three controls (D_1_, D_2 _and D_3_), all the trays were treated with 10 mg of fungus. Larvae were fed with Tetramin^® ^at a rate of 0.3 mg/larva/day. Mortality and pupation was taken into account for 10 days post treatment. The six treatments (D_1_, D_2 _and D_3_; both control and fungus treated) were replicated three times for both fungus and mosquito species. The replicates were spread over separate points in time.

### Statistical analysis

The effect of covariates, on the mortality rate of larvae, was analysed using Cox regression [33]. This model describes the increased or decreased likeliness of an event (in this case mortality) to occur, due to a covariate, in terms of hazard ratios (HR). All covariates were treated as time-independent. The p-values were adjusted for multiple comparisons within models and between analysis by comparing the Wald statistics with Chi-square distribution (at degrees of freedom equal to the number of covariates assessed in that model) and Bonferroni correction, respectively.

The model is based on the assumption of proportional hazard, which was tested graphically by plotting the cumulative hazard rates against time, stratified for each covariate. If the resulting curves showed growth in the same shape without crossing, the assumption was satisfied. The assumption was also checked by plotting the log-log survival functions obtained by Cox regression and Kaplan Meier (KM) analysis and confirmed if the resulting line showed a 45° trend [34]. Larvae that pupated were considered as censored.

Survival curves of different fungus concentrations, for each mosquito and fungus species, were compared pair-wise by KM analysis [35]. One-way ANOVA was employed to determine any significant difference in the wing lengths. All the analyses were performed using SPSS version 15 software (SPSS Inc. Chicago, IL, USA).

## Results

### Susceptibility of mosquito species, the effect of fungus species, larval stage and fungus concentration

**Figure 1:**
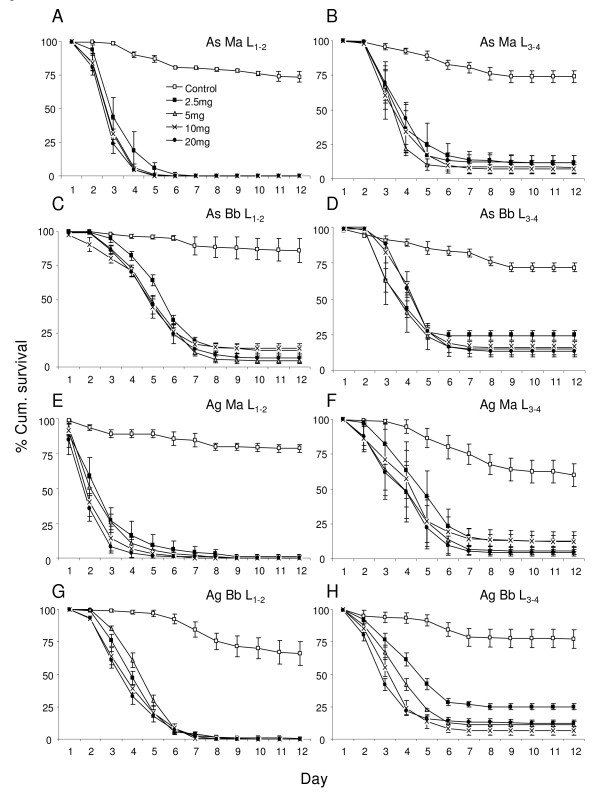
**Survival curves of mosquito larvae treated with different species and concentration of fungus**. Percentage cumulative survival curves of early (L_1-2_) and late (L_3-4_) larval stages of *An. stephensi *(*As*) and *An. gambiae *(*Ag*) when treated with different concentrations (2.5 mg, 5 mg, 10 mg and 20 mg) of *Metarhizium anisopliae *(*Ma*) and *Beauveria bassiana *(*Bb*). The survival curves include the larvae that survived due to pupation.

The HR's in Table 1A represent the hazard of *An. stephensi *larvae to die at different fungus concentrations as compared to *An. gambiae *larvae (reference species). As the values were not significant in any case, the results show that *An. stephensi *and *An. gambiae *are equally susceptible to both fungi at both early and late larval stage (Figure 1).

**Table 1:** Hazard ratios (95% CI) of mosquito larvae treated with different species and concentration of fungus

A. Mosquito spp.	Ma		Bb	
	L_1-2 _(A-E)	L_3-4_(B-F)	L_1-2_(C-G)	L_3-4_(D-H)
2.5	1.01 (0.63-1.62)	1.61 (1.07-2.43)	1.33 (0.82-2.17)	1.36 (0.86-2.16)
5	1.00 (0.66-1.69)	1.57 (1.04-2.37)	1.87 (1.15-3.03)	1.08 (0.69-1.69)
10	0.84 (0.52-1.34)	1.57 (1.04-2.36)	1.50 (0.92-2.44)	0.62 (0.40-0.98)
20	0.76 (0.48-1.22)	1.27 (0.84-1.90)	1.35 (0.83-2.19)	0.51 (0.32-0.80)

**B. Fungus spp.**	**Ag**		**As**	
	**L_1-2_(E-G)**	**L_3-4_(F-H)**	**L_1-2_(A-C)**	**L_3-4_(B-D)**

2.5	0.35 (0.22-0.54)**	1.18 (0.77-1.81)	0.28 (0.16-0.47)**	1.00 (0.64-1.55)
5	0.26 (0.17-0.41)**	1.15 (0.76-1.76)	0.24 (0.14-0.41)**	0.78 (0.51-1.20)
10	0.25 (0.16-0.38)**	1.56 (1.02-2.38)	0.24 (0.14-0.41)**	0.61 (0.40-0.95)
20	0.22 (0.14-0.35)**	1.65 (1.08-2.52)	0.22 (0.13-0.37)**	0.67 (0.43-1.03)

**C. Larval stage**	**Ag**		**As**	
	**Ma(E-F)**	**Bb(G-H)**	**Ma(A-B)**	**Bb(C-D)**

2.5	0.18 (0.12-0.28)**	0.57 (0.37-0.88)	0.23 (0.14-0.37)**	0.56 (0.33-0.93)
5	0.20 (0.13-0.31)**	0.85 (0.55-1.31)	0.22 (0.14-0.35)**	0.46 (0.27-0.76)
10	0.14 (0.09-0.22)**	0.83 (0.54-1.27)	0.20 (0.12-0.31)**	0.31 (0.18-0.52)**
20	0.14 (0.09-0.21)**	0.94 (0.61-1.44)	0.16 (0.10-0.26)**	0.31 (0.19-0.52)**

Mosquito species (*An. stephensi (As) *versus *An. gambiae (Ag)*), B) fungus species (*B. bassiana (Bb) *versus M. anisopliae (Ma)) and C) larval stage (Late (L3-4) versus early (L^1-2^)), for different concentrations (2.5, 5, 10, 20 mg/441 cm^2^) of the fungi. Asterisks indicate significance (P < 0.01) following adjustment for multiple comparison. Letters within parenthesis represent the graphs in Figure 1 that have been compared in the analysis.

In the case of fungus species, HR's in Table 1B show that the larvae that were exposed to *B. bassiana *had a lower mortality rate as compared to those exposed to *M. anisopliae *(reference species). However this was apparent only at the L_1-2 _stage of both *An. gambiae *and *An. stephensi *larvae. The HR's of L_3-4 _stages as compared to L_1-2 _stages (reference stage), in Table 1C show that at younger stage are more susceptible to fungal spores. This trend was clear in young larvae treated with *M. anisopliae*.

Kaplan Meier pair wise comparison showed all concentrations to be significantly different from their control (Figure 1, Table 2). Mostly the 2.5 mg treatment was significantly different from 5, 10, and 20 mg for both fungus species. In the case of *B. bassiana*, except for the L_1-2 _stage of *An. stephensi*, the 5 mg treatment was significantly different from the 10 and 20 mg treatments. Apart from the *B. bassiana *treatment of *An. gambiae *at the L_3-4 _stage, 10 and 20 mg treatments were not significantly different.

**Table 2:** Kaplan Meier pair-wise comparison of treatments (control, 2.5, 5, 10 and 20 mg)

Mosquito spp.	Larval stage	Fungus spp.	Treatment	Control	2.5	5	10
Ag	L_1-2_	Ma	2.5	***			
			5	***	ns		
			10	***	***	**	
			20	***	***	***	ns
		Bb	2.5	***			
			5	***	*		
			10	***	ns	***	
			20	***	*	***	ns
	L_3-4_	Ma	2.5	***			
			5	***	***		
			10	***	**	ns	
			20	***	***	ns	ns
		Bb	2.5	***			
			5	***	**		
			10	***	***	**	
			20	***	***	***	**
As	L_1-2_	Ma	2.5	***			
			5	***	***		
			10	***	***	ns	
			20	***	***	ns	ns
		Bb	2.5	***			
			5	***	**		
			10	***	**	ns	
			20	***	**	ns	ns
	L_3-4_	Ma	2.5	***			
			5	***	**		
			10	***	*	ns	
			20	***	ns	*	ns
		Bb	2.5	***			
			5	***	ns		
			10	***	**	***	
			20	***	***	***	ns

Early (L_1-2_) and late (L_3-4_) larval stages of *An. gambiae *(*Ag*) and *An. stephensi *(*As*) treated with *M. anisopliae *(*Ma*) or *B. bassiana *(*Bb*). *:*P *< 0.05, **: *P *< 0.01, ***: *P *< 0.001, ns = not significant.

For *M. anisopliae *the average percentage pupation in the control group of L_1-2 _*An. stephensi *was 73.5% while in the treated groups there was no pupation in all concentrations. In the L_3-4 _*An. stephensi *the control group had an average pupation of 74% and in the treatments it ranged from 7-11.5%. Similarly for *B. bassiana *treatments the average pupation in the control L_1-2 _*An. stephensi *was 86% while at different concentrations it ranged between 0-13%. For the L_3-4 _*An. stephensi *the control group had an average pupation of 72% while in the different *B. bassiana *treatments it ranged from 9 -24%. Further on for *M. anisopliae *treatments the average percentage pupation in the control group of L_1-2 _*An. gambiae *was 78.5% while in the treated groups it was 0-1% for different concentrations. The L_3-4 _*An. gambiae *control group had an average pupation of 60% while in the treatments it ranged from 4-12%. In case of *B. bassiana *treatments the average pupation in the control L_1-2 _*An. gambiae *was 77% while at different concentrations it ranged between 0-1%. For the L_3-4 _*An. gambiae *the control group had an average pupation of 66% while in the different *B. bassiana *treatments it ranged from 6.5 -25%.

### Effect of exposure time

**Figure 2:**
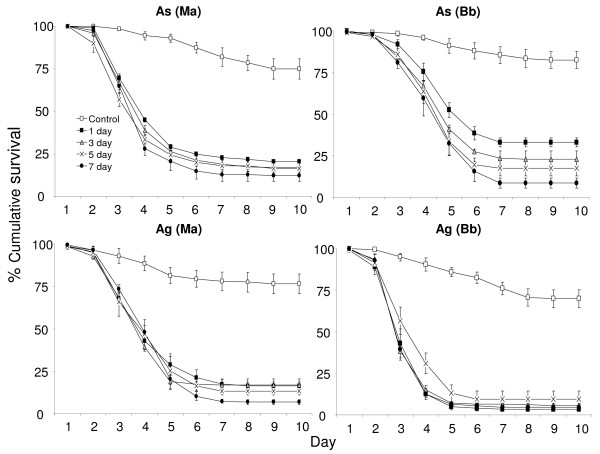
**Survival curves of mosquitoes for different exposure times**. Percentage cumulative survival curves of *An. stephensi *(*As*) and *An. gambiae *(*Ag*) larvae, exposed to *Metarhizium anisopliae *(*Ma*) and *Beauveria bassiana *(*Bb*) spores for 1, 3, 5 and 7 days. The survival curves include the larvae that survived due to pupation.

The larvae exposed to fungus for seven days were considered as the reference group. The HR's (Table 3), therefore, represent the increased or decreased hazard for the larvae when they were exposed to fungus (*M. anisopliae *and *B. bassiana*) for 0 (control), 1, 3 or 5 days as compared to the larvae exposed for 7 days. *Anopheles stephensi *and *An. gambiae *larvae exposed to fungus for 1, 3, and 5 days did not have a significant higher or lower hazard to larvae that were exposed for seven days. In other words 1, 3 and 5 days exposure to fungus had the same effect as seven days exposure (Figure 2). The control larvae were at a significantly lower hazard of dying as compared to the larvae exposed for seven days.

**Table 3:** Hazard ratios (95% CI) of mosquitoes for different exposure times

Mosquito spp.	Fungus spp.	Treatment Day(s)	HR (95% CI)
Ag	Ma	0	0.30 (0.22-0.42) *
		1	0.94 (0.74-1.19)
		3	1.14 (0.92-1.42)
		5	0.99 (0.79-1.24)
	Bb	0	0.12 (0.08-0.17)*
		1	0.97 (0.80-1.19)
		3	0.96 (0.79-1.17)
		5	0.79 (0.65-0.97)
As	Ma	0	0.19 (0.14-0.27)*
		1	1.01 (0.79-1.28)
		3	0.96 (0.75-1.23)
		5	1.11 (0.87-1.43)
	Bb	0	0.17 (0.12-0.25)*
		1	0.73 (0.58-0.92)
		3	0.81 (0.66-1.01)
		5	1.07 (0.86-1.32)

Hazard ratio (HR) of *An. gambiae *(*Ag*) and *An. stephensi (As) *larvae of the control (0 day) and treatments (exposed to *M. anisopliae *(*Ma*) or *B. bassiana *(*Bb*) spores for 1, 3 and 5 days) as compared to exposure for 7 days. * *P*< 0.05, adjusted for multiple comparison.

**Figure 3:**
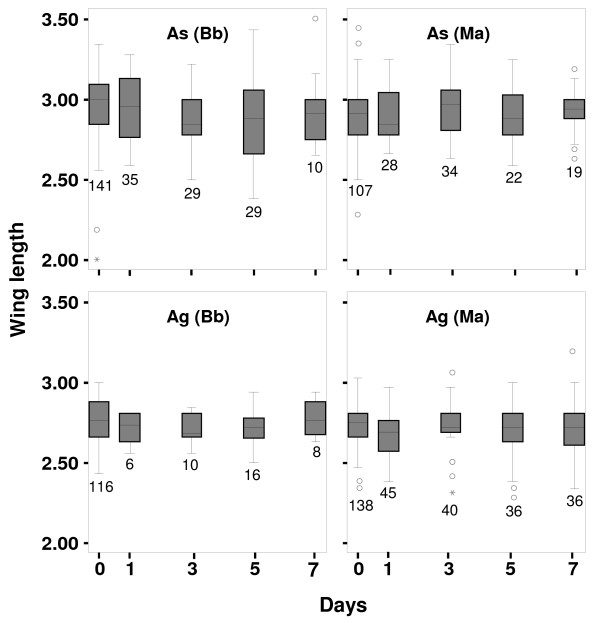
**Difference in wing lengths of adult mosquitoes**. Box plot of wing measurements (mm) for *An. stephensi *(*As*) and *An. gambiae *(*Ag*) larvae, exposed to *B. bassiana *(*Bb*) and *M. anisopliae *(*Ma*) spores for 1, 3, 5 and 7 days. Dots and stars represent outliers. Whiskers represent the range. Box limit, the 1^st ^(lower) and 3^rd ^(upper) quartiles and box dividing line, the median. The number of adults dissected is shown below each box.

There was no significant difference in the wing lengths of adults developing from larvae in the control and treated trays (1, 3, 5 and 7 days exposure) as shown in Figure 3. Some pupae and adults showed fungal infection. The fungus was confirmed as *M. anisopliae *or *B. bassiana *by microscopy. Table 4 shows the pupation and adult emergence (%) in each treatment and the percentage of larvae that showed infection at the pupal or adult stage e.g. 36 *An. stephensi *(*As*) pupae developed from the larvae exposed to *B. bassiana *spores for 5 days. Among those, four pupae and ten adults showed fungal infection. These ten adults account for nearly 35% of the total adults that made it to the adult stage.

**Table 4:** The number and percentage of pupae and adults that showed fungus infection

Mosquito spp.	Fungus spp.	Treatment (days)	Pupation		Pupae showing infection		Adult emergence		Adults showing infection	
			n	%*	N	%*	n	%*	n	%*
Ag	Ma	0	150	74.3	0	0	138	68.3	0	0
		1	52	26.5	0	0	45	23.0	3	1.5
		3	49	23.6	0	0	40	19.2	0	0
		5	42	21.2	0	0	36	18.1	5	2.5
		7	37	18.3	0	0	36	17.8	1	0.5
	Bb	0	127	70.6	0	0	116	64.6	0	0
		1	7	3.4	0	0	6	2.9	0	0
		3	11	5.5	0	0	10	5	1	0.5
		5	19	8.9	0	0	16	7.5	5	2.3
		7	9	4.3	1	0.5	8	3.9	0	0
As	Ma	0	153	72.5	0	0	107	50.7	0	0
		1	52	25.3	2	1	28	14.1	4	2
		3	48	24.9	2	1	34	17.7	9	4.6
		5	40	22.2	3	1.7	22	13.3	7	3.9
		7	38	19.7	1	0.5	19	11.1	1	0.5
	Bb	0	159	82	0	0	141	72.7	0	0
		1	66	34.9	6	3.2	35	18.5	5	2.6
		3	51	24.9	10	4.9	29	14.1	8	3.9
		5	36	17.6	4	2	29	14.1	10	4.9
		7	17	8.5	2	1	10	5	1	0.5

*An. gambiae (Ag) *and *An. stephensi *(*As*) that developed from the larvae (n = 200) treated with fungus (*M. anisopliae *(*Ma*) or *B. bassiana *(*Bb*)) spores. * Percentages are based on total number of larvae treated.

**Table 5:** Hazard ratios (95% CI) of mosquitoes provided with different quantities of food

Mosquito spp.	Fungus spp.	Food provision treatment	HR (95% CI)
Ag	Ma	0.5	5.25 (2.97-9.25)^a^
		0.3	4.30 (3.24-5.69)^a^
		0	1.98 (1.59-2.46)^b^
	Bb	0.5	4.40 (2.88-6.73)^a^
		0.3	6.73 (4.79-9.45)^a^
		0	2.57 (2.07-3.20)^b^
As	Ma	0.5	5.25 (2.97-9.25)^a^
		0.3	7.04 (5.02-9.87)^a^
		0	1.90 (1.54-2.34)^b^
	Bb	0.5	19.21 (9.45-39.03)^a^
		0.3	5.05 (3.56-7.16)^b^
		0	1.22 (1.00-1.49)^c^

Hazard ratio (HR) of treated (10 mg, *M. anisopliae *(*Ma*) or *B. bassiana *(*Bb*)) *An. gambiae *(*Ag*) and *An. stephensi *(*As*) larvae compared to untreated larvae provided with the same quantity of food (0.5, 0.3 or 0 mg/larva/day). HR's with letters in common indicate overlapping of 95% confidence limits.

**Table 6:** Hazard ratios (95% CI) of mosquitoes at different larval densities

Mosquito spp.	Fungus spp.	Larval density	HR (95% CI)
Ag	Ma	0.1	11.87 (6.62-21.31)^a^
		0.3	4.37 (3.06-6.23)^b^
		0.5	3.73 (3.07-4.59)^b^
	Bb	0.1	3.13 (2.15-4.54)^a^
		0.3	8.39 (6.40-10.10)^b^
		0.5	29.32 (20.72-41.48)^c^
As	Ma	0.1	15.12 (8.42-27.16)^a^
		0.3	61.13 (27.30-136.89)^b^
		0.5	73.66 (34.98-155.11)^b^
	Bb	0.1	13.88 (7.44-25.89)^a^
		0.3	47.7 (21.27-106.99)^a,b^
		0.5	63.23 (29.97-133.41) ^b^

Hazard ratios (HR) of *An. gambiae *(*Ag*) and *An. stephensi *(*As*) larvae treated with *M. anisopliae *(*Ma*) or *B. bassiana *(*Bb*) spores compared to un-treated larvae at the same density (0.1, 0.3 or 0.5 larvae/cm^2^). HR's with letters in common indicate overlapping of 95% confidence limits.

### Effect of food quantity

**Figure 4:**
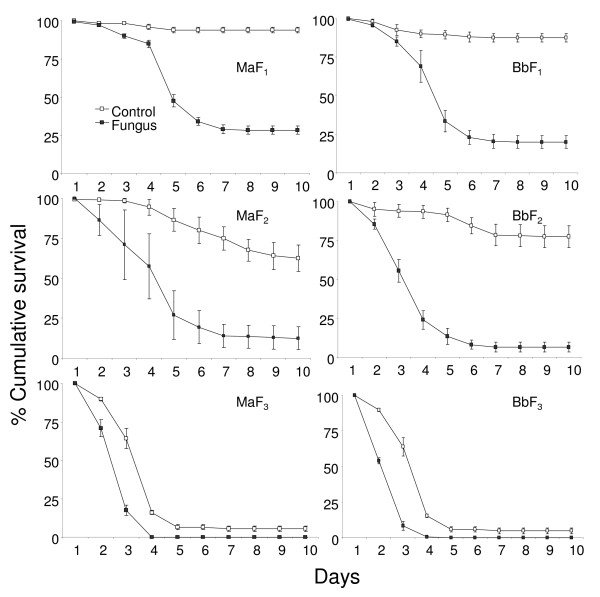
**Survival curves of *An. gambiae *provided with different quantities of food**. Percentage cumulative survival curves of *An. gambiae *larvae, exposed to 10 mg of *M. anisopliae *(*Ma*) and *B. bassiana *(*Bb*) spores, with different food quantities (F_1_, 0.5 mg; F_2_, 0.3 mg; F_3_, no food). The survival curves include the larvae that survived due to pupation.

**Figure 5:**
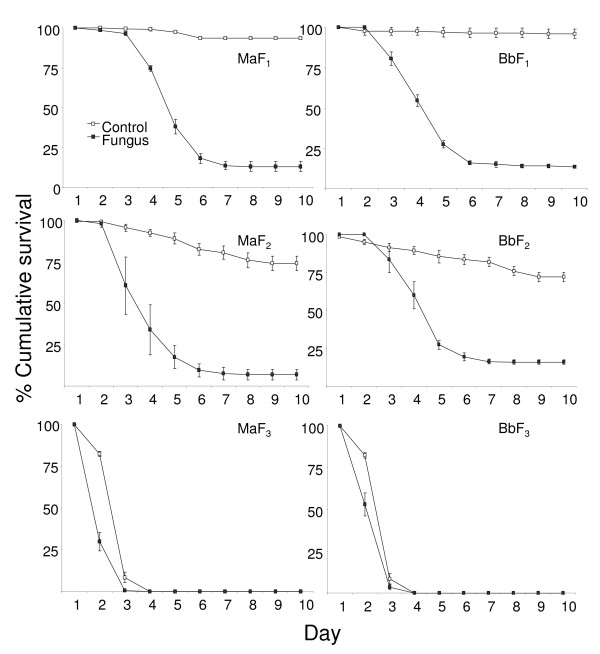
**Survival curves of *An. stephensi *provided with different quantities of food**. Percentage cumulative survival curves of *An. stephensi *larvae, exposed to 10 mg of *M. anisopliae *(*Ma*) and *B. bassiana *(*Bb*) spores, with different food quantities (F_1_, 0.5 mg; F_2_, 0.3 mg; F_3_, no food). The survival curves include the larvae that survived due to pupation.

Table 5 represents the hazard for larvae treated with fungal spores as compared to untreated larvae (control) provided with the same quantity of food (0.5, 0.3 or 0 mg/larva/day). The HR's show that the hazard decreased with decreasing food quantity (Figures 4 and 5). In the fungal treatment where larvae had no food the hazard was 1-2 times higher than that for their control. The larvae that had food available were 4-19 times more likely to acquire a lethal infection than their control counterparts. The overlapping 95% confidence interval (Table 5) indicates no difference in the hazard of larvae provided with 0.3 or 0.5 mg of food. The HR's were higher in the presence of food rather than in the absence. In the absence of food 100% of the control larvae died within 3-4 days. The fungal spores kill slowly so by the time the infected larvae died due to both ingested spores and absence of nutrients, the control starving larvae also started to die. This led to a low hazard ratio. In the other two treatments (0.3 and 0.5 mg/larva/day) the HR's were high because the survival of the fed-control larvae was also high. The overlapping 95% confidence interval (Table 5) indicates no difference in the hazard of larvae provided with 0.3 or 0.5 mg of food.

### Effect of larval density

**Figure 6:**
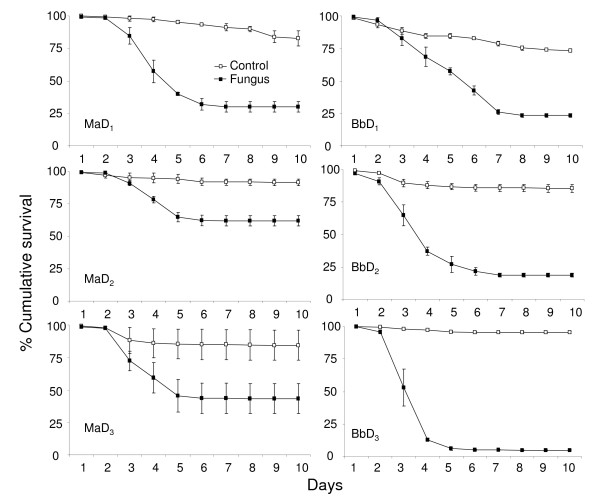
**Survival curves of *An. gambiae *at different densities**. Percentage cumulative survival curves of *An. gambiae *larvae, exposed to 10 mg of *M. anisopliae *(*Ma*) and *B. bassiana *(*Bb*) spores, at different densities (D_1_, 0.5 larvae/cm^2^; D_2_, 0.3 larvae/cm^2^; D_3_, 0.1 larvae/cm^2^). The survival curves include the larvae that survived due to pupation.

**Figure 7:**
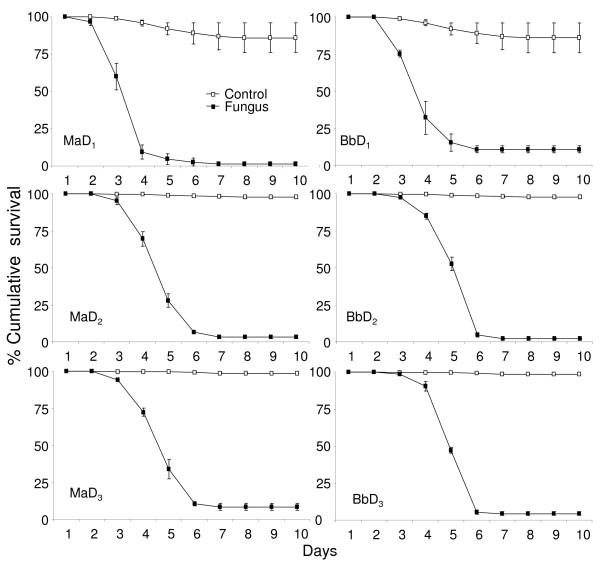
**Survival curves of *An. stephensi *at different densities**. Percentage cumulative survival curves of *An. stephensi *larvae, exposed to 10 mg of *M. anisopliae *(*Ma*) and *B. bassiana *(*Bb*) spores, at different densities (D_1_, 0.5 larvae/cm^2^; D_2_, 0.3 larvae/cm^2^; D_3_, 0.1 larvae/cm^2^). The survival curves include the larvae that survived due to pupation.

Based on the fact that infection, caused during the first 1-2 days of fungus treatment, accounts for most of the mortality, density was considered a time-independent covariate. For each density, the control was considered as the reference i.e. the HRs in Table 6 represent the hazard of the larvae treated with fungus compared to untreated larvae at the same density. Except for the *M. anisopliae *treatment of *An. gambiae *the HRs increased with an increase in density (Figures 6 and 7). Increase in HRs indicates that with increasing larval density the mortality rate increases. However, the overlapping 95% CI's (Table 6) indicate that, although such trends exist, this was not significant in all cases.

## Discussion

This study shows the susceptibility *An. stephensi *and *An. gambiae *larvae to *M. anisopliae *and *B. bassiana *and how larval stage, fungus species and fungus concentration can influence the mortality rate. In addition, it also provides an insight into the impact of exposure duration, quantity of food available, and larval density on the mortality rate.

Both mosquito species were equally susceptible to *M. anisopliae *and *B. bassiana *spores. *M. anisopliae *and *B. bassiana *spores act as midgut toxins and enter the body mainly through the mouth. When applied over the water surface they are readily available to the larvae of *An. stephensi *and *An. gambiae *as well as other anopheline species, which are surface-feeders. Because anophelines have similar filtering and ingestion rates both *An. stephensi *and *An. gambiae *were equally affected [18,36-40].

The lower hazard of *B. bassiana *spores as compared to *M. anisopliae *spores at the L_1-2 _stage cannot be due to the spore size as in this regard *M. anisopliae *(2.5-3.5 μm) and *B. bassiana *(2-3 μm) are comparable and within the range of particle size appropriate for anopheline collecting-filtering feeding mode (0.45 μm -1 mm) [15,38]. However, the difference in the rapidity of vegetative growth found in *M. anisopliae *and *B. bassiana *may cause the difference in the observed effect of the two fungi. *Metarhizium anisopliae *is characterized by a rapid vegetative growth although at the expense of sporulation, while *B. bassiana *has a slow vegetative growth with high total sporulation [41]. Slow vegetative growth may be associated with the slow release of endo-toxins inside the larval body and, thus, a delayed mortality.

The reason for the difference in lethal effects of *M. anisopliae *and *B. bassiana *at the younger (L_1-2_) stage and the reduced susceptibility of the older (L_3-4_) stage can be considered together. The younger stage has a longer developmental time ahead until pupation, during which they feed and moult two to three times. Larvae are more vulnerable to infection immediately after ecdysis because of the soft cuticle. Younger larval stages thus have an increased probability of acquiring an infection. Late larval stages, on the other hand, have reduced food intake and a thicker cuticle and thus it is less likely that spores would enter or penetrate their body [16,42,43].

Dry spores were used and dusted on the water surface because they are more effective than the normal laboratory formulations (0.1% Tween 80 solution) that reduce the clumping but cause the spores to sink as the water surface is the main foraging site of *Anopheles *larvae [18,38,44]. Although there was a difference in the effect of fungal concentrations, this was not proportional. The fungal spores are hydrophobic and when applied over the water surface without a surfactant they clump together into masses that become dense over time. Larvae may have rejected the spore mass as food most likely because of large clump size and the density of the mass made spore attachment avoidable. As a result, high concentrations did not provide a better spatial and temporal coverage that could have resulted in increased mortality rate. To achieve the full benefits of biological control it is better to use fungal spores rather than extracted endotoxins [45]. When spores enter the larval body through the mouth or siphon they mechanically block these passages while a few attach to the interior. The attached spores germinate releasing endotoxins as well as damaging the larval tissues with their vegetative growth [46]. In this case there is a whole spectrum of offence that has to be tackled by the larval immune system. The more variable the modes of action, the lower is the probability that resistance will develop against the control agent [47].

The exposure time experiment showed that one day exposure to fungus spores has more or less the same effect as an exposure of seven days. One explanation would be the clumping of spores over time meaning that the spore coverage and thus its effect is not the same over time. Another explanation may be the ingestion rate of larvae. Normally food passes through the larval guts in 1-1.5 hours [37,38]. Within 24 hours of exposure, swollen (ready to germinate) spores can be detected in the gut and even in exuviae. Although mortality occurs after 4 days, it takes only one day for the spores to penetrate the tissues [30]. As a result, even if the larvae are shifted to un-treated water after a day they carry a sufficient amount of spores required for a fatal infection. This is useful as in field application dry spores may loose their virulence within days due to environmental conditions (notably UV radiation).

Although the larvae exposed to *B. bassiana *had a lower mortality rate as compared to the ones exposed to *M. anisopliae*, the proportion of larvae that made it to the adult stage was equal. As a result, if used, both fungi will equally contribute to reducing malaria transmission. For a better concentration effect it is important to have a formulation that spreads the spores while leaving them at the surface. It will not only facilitate the application but also decrease the required amount of spores. Considering the entry routes of spores into the larval body, it is important to have an organic and dry formulation for the control of anopheline species [18,41]. The formulation is also necessary to increase the persistence of the fungal spores. Fungal spores are sensitive to temperature, humidity and ultraviolet radiation. High relative humidity triggers germination in the spores and is therefore likely to play a negative role when spores are applied over the water surface [48,49]. Although one-day exposure is enough to cause significant mortality, the lack of a residual effect is a big disadvantage. This can possibly be solved by selection for tolerant isolates and formulations [22,48-51].

In some treatments up to 9% of the total fungus-infected larvae developed into pupae or adults that showed fungus infection. The passage of infection from the larvae to pupae and/or adult was also observed by Lord *et al*, Wilson *et al *and Sandhu *et al *[16,19,52]. This study reports the transfer of *M. anisopliae *and *B. bassiana *infection from the larvae to the pupae and adults of both *An. stephensi *and *An. gambiae*. Studies that deal with the immune system of insects during metamorphosis show increased anti-bacterial activities [53,54]. However, there is not much information about anti-fungal activity. Extracts from the medicinal plant, *Leuzea carthamoides*, known to contain 20-hydroxyecdysone showed anti-bacterial effect but in the study anti-fungal effects were not tested [55]. In another study juvenile hormone (JH) showed no effect on fungal growth [56]. The transfer of fungal infection may be due to the absent or moderate anti-fungal activity during metamorphosis. This ability of the fungus, to transfer from one life-cycle stage to the other, through the extreme histological changes during metamorphosis is very interesting as fungal exposure at a late larval stage can result in adults with reduced longevity.

At the tested concentration (10 mg) of fungal spores, the hazard increased with increase in larval density. The increasing hazard might be the result of increased larval mobility, which in turn increases the chance of contacting fungus spores and causing stress due to competition for space. At a low density, on the other hand, larvae have a reduced chance of contacting fungus spores because of less motility. However, it might be so that with a lower fungus concentration the larvae at higher density show low mortality due to reduced spore-share per larva [57]. The concentration of fungus is therefore critical. The difference in the *M. anisopliae *treatment of *An. gambiae *is probably because of more L_4 _larvae in the cohort as by the 3^rd ^day pupation was already 40% in D_2 _and 30% in D_3 _fungus-treated trays.

In the laboratory both fungal species show potential as larval control agents. To check their aptitude in the field, trials need to be conducted under natural conditions where, apart from the persistence and efficacy of the spores, the fungal species need to be observed for their non-target effect. Non-target effects of *M. anisopliae *and *B. bassiana *have been reported in aquatic environments [58-60]. However, these tests were done at extreme circumstances, which are highly unlikely under field conditions as fungal spores themselves are sensitive to environmental factors [46,48,49]. It is therefore important to test the non-target effects of these fungi in more realistic settings. As far as humans are concerned clinical cases have been rare and associated with mass exposure and immunodeficiency [15,61]. Thus, although any adverse effects on human are highly unlikely studies need to be done on formulations and delivery methods that reduce the chances of human and fungal contact.

## Conclusions

It is clear that both *M. anisopliae *and *B. bassiana *are highly effective in reducing larval survival and adult emergence. At times when insecticide resistance is increasing it is important to have a wider option of new biological and environmentally friendly control agents. However studies need to be carried out to develop efficient formulations and delivery methods for these fungi. Fungi might than present a new generation of larval control agents.

## Competing interests

The authors declare that they have no competing interests.

## Authors' contributions

TB and AM carried out the experimental work; TB performed the statistical analysis and drafted the manuscript. CJMK helped with the statistical analyses and drafting the manuscript. WT provided scientific guidance in interpretation of the findings. BGJK provided overall supervision in the study design, coordination and drafting of the manuscript. All authors read and approved the final manuscript.
